# Prediction of GTP interacting residues, dipeptides and tripeptides in a protein from its evolutionary information

**DOI:** 10.1186/1471-2105-11-301

**Published:** 2010-06-03

**Authors:** Jagat S Chauhan, Nitish K Mishra, Gajendra PS Raghava

**Affiliations:** 1 Bioinformatics Centre, Institute of Microbial Technology (IMTECH), Sector 39-A, Chandigarh -160036, India

## Abstract

**Background:**

Guanosine triphosphate (GTP)-binding proteins play an important role in regulation of G-protein. Thus prediction of GTP interacting residues in a protein is one of the major challenges in the field of the computational biology. In this study, an attempt has been made to develop a computational method for predicting GTP interacting residues in a protein with high accuracy (Acc), precision (Prec) and recall (Rc).

**Result:**

All the models developed in this study have been trained and tested on a non-redundant (40% similarity) dataset using five-fold cross-validation. Firstly, we have developed neural network based models using single sequence and PSSM profile and achieved maximum Matthews Correlation Coefficient (MCC) 0.24 (Acc 61.30%) and 0.39 (Acc 68.88%) respectively. Secondly, we have developed a support vector machine (SVM) based models using single sequence and PSSM profile and achieved maximum MCC 0.37 (Prec 0.73, Rc 0.57, Acc 67.98%) and 0.55 (Prec 0.80, Rc 0.73, Acc 77.17%) respectively. In this work, we have introduced a new concept of predicting GTP interacting dipeptide (two consecutive GTP interacting residues) and tripeptide (three consecutive GTP interacting residues) for the first time. We have developed SVM based model for predicting GTP interacting dipeptides using PSSM profile and achieved MCC 0.64 with precision 0.87, recall 0.74 and accuracy 81.37%. Similarly, SVM based model have been developed for predicting GTP interacting tripeptides using PSSM profile and achieved MCC 0.70 with precision 0.93, recall 0.73 and accuracy 83.98%.

**Conclusion:**

These results show that PSSM based method performs better than single sequence based method. The prediction models based on dipeptides or tripeptides are more accurate than the traditional model based on single residue. A web server "GTPBinder" http://www.imtech.res.in/raghava/gtpbinder/ based on above models has been developed for predicting GTP interacting residues in a protein.

## Background

Many proteins such as protein kinase, G-protein, dehydrogenase enzymes, Ras group of proteins and Src group proteins bind to nucleotide (adenine and guanine) for their function [1,2]. These proteins play an important role in cellular transport mechanisms, cell signaling, muscle contraction and cellular motility. At present the number of known protein structures has increased enormously due to rapid advancement in the structural genomics projects. Protein Data Bank (PDB), a representative database of biomolecular structures contains about 64000 experimentally determined protein structures including different types of ligands. The gap between the number of reported sequences and experimental structures continues to increase. Finding and predicting nucleotide-binding residues in a protein structures are important for understanding the function of these proteins. Previous studies on protein nucleotide interactions show that these proteins have binding site of specific characteristics. For example, Walker's A motif, a motif present in P-loop [3,4], has been proposed for phosphate binding site. But this motif is not sufficient for identification of all GTP interacting residues. Many nucleotide-binding proteins share common features but molecular recognition studies show that adenine and guanine ligand binding site in these protein are different, in terms of binding site amino acid propensities and propensities to form hydrogen bonds to the bases [5,6].

So there is a need to develop alternate technique, such as computational techniques for predicting ligand-interacting residues in a protein. Broadly, the existing method of predicting function of a protein can be divided in two categories; I) protein level prediction, where function of whole protein is predicted [7-9] and II) residue level prediction where function of each residue in a protein is predicted [10-13]. In this study, we have used the second approach for predicting GTP interacting residues in a protein from its amino acid sequence. The GTP ligand is crucial for various protein receptors for activation of enzymatic reaction. Mutations in the GTP binding site alter the biochemical reaction and reduce GTPase activity [14]. Thus the GTP binding site will act as potential site for docking studies. Identification of GTP interacting residues from its amino acid sequence is very important for researchers working in the filed of drug discovery. During last few years, many nucleotides (GTP) binding proteins have been discovered due to advancement in sequence technology. This poses a challenge for bioinformaticians to identify GTP interacting residues in newly sequenced GTP binding proteins; identification of GTP interacting techniques is time consuming and costly. To the best of our knowledge, no sequenced based method has been developed so far for predicting GTP interacting residue in a protein.

In this study, we have used two powerful machine-learning techniques, Artificial Neural Network (ANN) and Support Vector Machines (SVM), for developing prediction models. First, ANN based method developed using amino acid sequence and evolutionary information. Secondly, we used SVM based technique, which has been used for developing various bioinformatics methods in the past like predicting protein subcellular location [7], protein structure class [9], specificity of GalNAc-transferase [15], protein-ligand interacting residues [16]. It has been shown in number of studies that SVM perform better than ANN [17,18]. In existing residue level prediction methods, a pattern is generated to predict function of a central residue - for example, a pattern/window of 17 residues contains query residue, 8 residue left and 8 residue right of query residue. In this study, for the first time, we have introduced new concept for generating patterns for predicting dipeptide (two consecutive GTP interacting residues) and tripeptide (three consecutive GTP interacting residues) from a single pattern.

## Results

### Composition analysis

**Figure 1:**
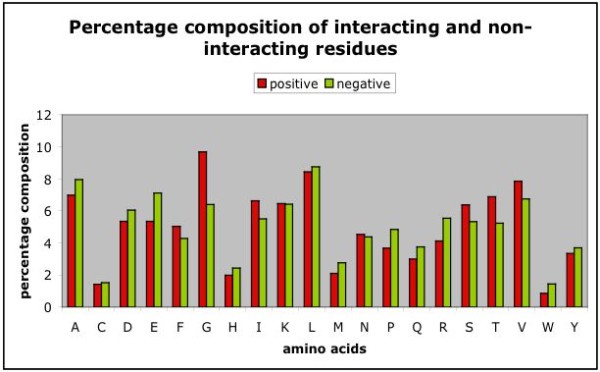
**Composition of GTP interacting and non-interacting residues**. Here positive means GTP-interacting position and negative means non-interacting residues.

We have analyzed the composition of GTP interacting and non-interacting residues in GTP binding proteins and observed that certain types of residues are preferred in GTP interaction. As shown in Figure 1, composition of residues (e.g., Gly, Ile, Ser, Val, Thr) involved in GTP-interaction is significantly higher. Earlier nucleotide-binding proteins studies [3,4] showed that these proteins having a motif [GXXXXGK(T/S)] in which G, K, T and S amino acids are conserved amino acid residues, so these amino acid residues are comparatively higher in GTP interacting site.

### Concept of amino/di/tripeptide

All existing interacting residue prediction method uses single residue based technique (SRT), where patterns are generated and discriminated to predict central residue of a pattern (see Materials and Methods). We have also implemented same SRT in this study, where we have generated 876 positive patterns (GTP interacting central residue) from 44 non-redundant protein chains. In this study, we have introduced two new concepts for predicting GTP interacting residues using dipeptide based technique (DPT) and tripeptides based technique (TPT). In case of DPT, we have generated 451 positive patterns where central dipeptide (two consecutive residues) is GTP interacting (see Materials and Methods). In case of TPT, we have generated 256 positive patterns where central tripeptide (three consecutive residues) is GTP interacting. Figure S1 (Additional file 1) shows the above concept visually.

### Artificial Neural Network based Models

We have develop ANN based model on main dataset using learning parameter 0.01. We have achieved maximum MCC of 0.24 with accuracy 61.30% using SRT (Table 1). It has been shown in the past that evolutionary information provides more information than single sequence [19,20]. Thus, we have also developed ANN based models using evolutionary information in the form of PSSM profiles. The performance of our PSSM based improved significantly from MCC 0.24 to 0.39 (Table 2).

**Table 1:** The performance of ANN models using single residue based technique (SRT) from amino acids sequence of protein.

Threshold	Sensitivity	Specificity	Accuracy	MCC
0	100	0	50.00	0
0.1	59.02	63.93	61.47	0.23
**0.2**	**45.78**	**76.83**	**61.3**	**0.24**
0.3	36.76	83.68	60.22	0.23
0.4	29.11	89.04	59.08	0.23
0.5	23.29	91.89	57.59	0.21
0.6	18.15	94.75	56.45	0.2
0.7	12.79	97.37	55.08	0.19
0.8	08.11	98.74	53.42	0.16
0.9	03.42	99.54	51.48	0.11
1.0	0	100	50.00	0

**Table 2:** The performance of ANN model using single residue based technique (SRT) from evolutionary information of protein (PSSM profile).

Threshold	Sensitivity	Specificity	Accuracy	MCC
0	100	0	50.00	0
0.1	65.33	70.48	67.91	0.36
**0.2**	**58.58**	**79.18**	**68.88**	**0.39**
0.3	53.89	82.95	68.42	0.39
0.4	50.00	86.04	68.02	0.39
0.5	46.80	88.33	67.56	0.39
0.6	42.56	90.27	66.42	0.37
0.7	39.02	92.11	65.56	0.37
0.8	32.49	95.08	63.79	0.35
0.9	22.88	97.03	59.95	0.3
1.0	0	100	50	0

### SVM based Models

#### Single Residue based Technique (SRT)

**Figure 2:**
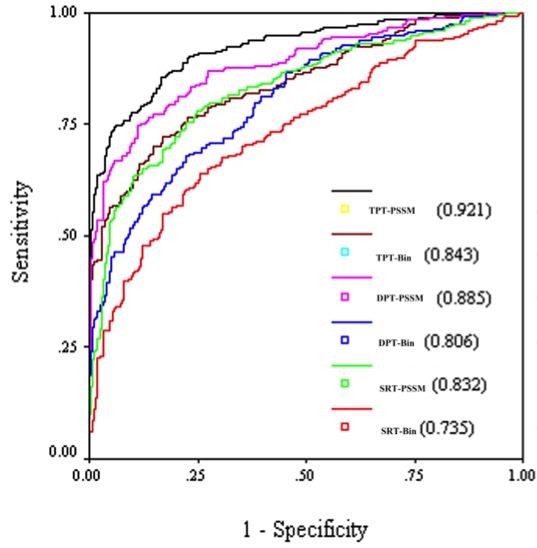
**ROC plots for various models**. SRT-Bin, DPT-Bin and TPT-Bin shows SVM models based on SRT, DPT and TPT respectively, using single sequence. Similarily SRT-PSSM, DPT-PSSM and TPT-PSSM show SVM models based on SRT, DPT and TPT respectively, using PSSM profile). AUC corresponding to each model is shown in bracket.

It has been shown in the past that SVM is a powerful technique for classification. Thus, we have also developed SVM models using SRT for predicting GTP interacting residues in a protein from their primary sequence. As shown in Table 3, we have achieved MCC 0.37 with precision 0.73, recall 0.57, accuracy 67.98%, and F1 score 0.64. The performance of the model in the form of ROC plot is shown in Figure 2. We have achieved AUC 0.735 using SVM model for window length 17 residues. These results demonstrate that SVM models perform better than ANN based models in prediction of GTP interacting residues. We have observed that PSSM based ANN models perform better than sequence based ANN models (Table 1 &2). Thus we have developed PSSM based SVM models for predicting GTP interacting residues in proteins from their evolutionary information. We computed a vector of dimension of 357 from PSSM matrix (See Methods). Finally, a SVM model was developed using PSSM and we achieved MCC of 0.55 with precision 0.80, recall 0.73, accuracy 77.17% and F1 score 0.76 (Table 3). The performance of PSSM based model in term of AUC also increased to 0.832 (Figure 2). These results clearly indicate that evolutionary information is important for the prediction of GTP interacting residues.

**Table 3:** The performance of SVM models on main dataset using using SRT, DPT and TPT (See Additional file 1 Table S1-6 for detail)

Types of patterns	Method	Sensitivity (Recall)	Specificity	Accuracy	Precision	MCC	F1 Score
SRT	Single Sequence	57.19	78.77	67.98	0.73	0.37	0.64
	PSSM profile	73.23	81.12	77.17	0.80	0.55	0.76

DPT	Single Sequence	60.31	86.25	73.28	0.81	0.48	0.69
	PSSM profile	73.61	89.14	81.37	0.87	0.64	0.80

TPT	Single Sequence	62.50	89.06	75.78	0.85	0.53	0.72
	PSSM profile	73.44	94.53	83.98	0.93	0.70	0.82

Where SRT: Single Residue based Technique

DPT: Dipeptide based Technique

TPT: Tripeptide based Technique

#### Dipeptide based Technique (DPT)

All the above models were based on SRT where patterns are generated and classified for predicting function of a single central residue of a pattern. In this study, we have generated and classified pattern for predicting function of two central residues of a pattern called DPT. First we have generated patterns of length 16 residues which contained either GTP interacting or non-interacting central dipeptide. Total 451 positive patterns with two central interacting residues (dipeptides) were obtained. Finally we have developed SVM model using DPT for predicting GTP interacting in a proteins from its amino acid sequence and achieved maximum MCC 0.48 with precision 0.81, recall 0.60, accuracy 73.28% and F1 score 0.69. In order to improve the performance of SVM models using DPT, we have developed PSSM based models instead of single sequence based model and achieved MCC 0.64 with precision 0.87, recall 0.74, accuracy 81.37% and F1 score 0.80 (Table 3).

### Tripeptide based Technique (TPT)

**Figure 3:**
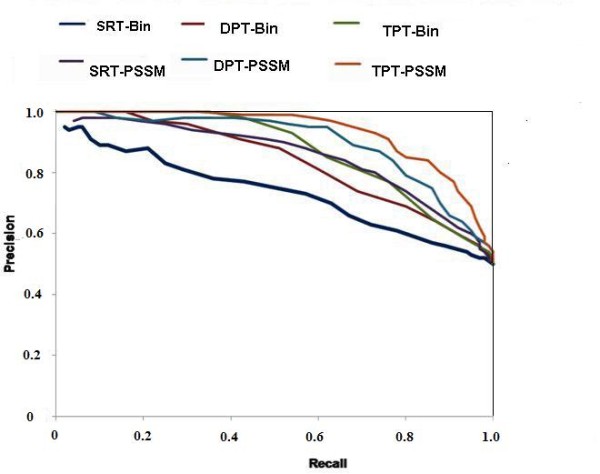
**Precision/Recall curves (PR curve) of different patterns**. Where SRT is Single Residue interacting patterns, DPT is Dipeptide (two consecutive amino acid residues) interacting patterns and TPT is Tripeptide interacting patterns in fixed window length amino acid patterns. Bin is Binary patterns.

It has been shown above that the models using DPT are more accurate than models using SRT. We thus extended our approach to tripeptides. In this case, the patterns are generated and classified for predicting GTP interacting central tripeptide (three consecutive residues) of a pattern of length 17 residues. We obtained 256 positive patterns where central tripeptides was GTP interacting. Finally SVM models using TPT were developed for predicting GTP interacting tripeptides in proteins from their amino acid sequence. We have achieved MCC 0.53 with precision 0.85, recall 0.63, accuracy 75.78% and F1 score 0.72 using single sequence. In term of AUC we achieved 0.843 (Figure 2). In addition to single sequence, we have also developed PSSM based SVM models using TPT and achieved MCC 0.70 with precision 0.93, recall 0.73 and accuracy 83.98% and AUC 0.921. In this work, we have also provided precision/recall (PR) curve, which is widely used informative picture to evaluate the performance of prediction method and discriminate them (Figure 3).

### Performance of SVM on Realistic dataset

All the above models were developed on main dataset where positive and negative pattens are nearly same. Though equal number of positive and negative patterns is important for learning any classification technique. In reality, a GTP binding protein has only few GTP interacting residues. The question arises whether models developed on equal number of negative and positive patterns are valid on real situation. Thus, there is also a need to develop models based on realistic data where negative patterns are much more than positive patterns. First, we have develop SVM model using SRT on a realistic dataset (contains 876 positive and 16831 negative patterns) and achieved MCC 0.38. The performance increased to MCC 0.57 when we used PSSM profile instead of single sequence. Though performance on realistic dataset is lower than performance on main dataset but trend is same. Similarly, we have developed SVM models using DPT and TPT and achieved MCC 0.42 and 0.44 on realistic data. In case of DPT and TPT, realistic contains 10 times negative patterns than positive patterns. The performance of PSSM based SVM models using DPT and TPT was 0.69 and 0.75 on realistic dataset (Table 4).

**Table 4:** The performance of SVM model on realistic dataset using SRT, DPT and TPT (See Additional file 1 Table S7-12 for detail)

Types of patterns	Method	Sensitivity (Recall)	Specificity	Accuracy	Precision	MCC	F1 Score
SRT	Single Sequence	24.00	99.36	95.65	0.66	0.38	0.36
	PSSM profile	41.65	99.55	96.57	0.83	0.57	0.56
DPT	Single Sequence	51.22	93.52	89.65	0.44	0.42	0.48
	PSSM profile	62.31	98.63	95.33	0.82	0.69	0.71
TPT	Single Sequence	50.39	94.60	90.61	0.48	0.44	0.49
	PSSM profile	66.80	99.11	96.30	0.88	0.75	0.76

Where SRT: Single Residue based Technique

DPT: Dipeptide based Technique

TPT: Tripeptide based Technique

## Discussion

The GTP interacting proteins play a vital role in signaling pathways, in which GTP is used as a substrate by kinases that phosphorylate proteins. Finding and predicting GTP-binding residues in protein are important subjects in protein interaction studies. The identification of GTP interacting residues is difficult by using in-vitro techniques, so there is a need for computational method to identify GTP binding sites on the basis of protein sequence. In the past, the structural based method for predicting GTP binding site using empirical scores system was developed [21]. This method detects GTP binding sites with low accuracy but do not provide information about GTP interacting residues. There is no method which can predict GTP-interacting residues in a protein using their amino acid sequence.

In this study, we have analyzed GTP interacting residues and its neighbors, and found that there is a significant difference in interacting and non-interacting residues. In this method, first we considered single GTP interacting residue, then we introduced new concepts of predicting GTP interacting dipeptide (two consecutive GTP interacting residues) and tripeptide (three consecutive GTP interacting residues). First, we have develop a neural network based method for predicting GTP interacting residues but its performance was poor. The earlier method shows that SVM performs better than any other artificial intelligence technique in predicting interacting residues. We have developed SVM models based on binary patterns of single GTP interacting residue, GTP interacting dipeptide and GTP interacting tripeptide. It has been shown in number of previous studies that the models based on evolutionary information are more accurate than models based on single sequence [10,16]. Thus, we have also developed SVM model based on evolutionary information (PSSM profiles) and observed that SVM models based on evolutionary are more accurate than SVM models based on single sequence.

One of the novelties of this study is the application of new strategy for prediction. We have used a pattern to predict dipeptide or tripeptides instead of single residue. Our models based on dipeptide or tripeptides are much more accurate than models based on single residue.

One of the major problems is selection of threshold that same MCC and F1 score may be obtained for different threshold (Additional file 1 Table S1 to S12). This raises a question which threshold score one should select as each one has different combination of sensitivity, accuracy and precision. In this study, we have varied threshold in the range of -1 to +1. Normally we select those thresholds where maximum values are maximum in the following order MCC, accuracy and precision with least difference in sensitivity and specificity. But this criteria is not always achievable, because the higher the recall, the lower the precision and vice versa. In this work, when we have used main dataset, there is least difference between recall and specificity with maximum MCC at specific threshold, but in realistic datasets we have achieved maximum MCC where recall was very less than specificity at specific threshold. Finally, we have computed the area under the ROC curve (AUC) for further analyzing the significance of the results (Figure 2). The ROC curve have been generated by varying threshold score, it has been observed that evolutionary information was important for the prediction of GTP interacting residues.

This study raises a question whether models should be developed based on SRT or DPT or TPT. As we know SRT is almost a standard in the filed and used in all studies, It's major advantage is that it does not bother the distribution of GTP interacting residues so it cover all residues. In case of DPT and TPT, coverage will depend on distribution of residues. In this study, we have obtained 876, 451 and 256 examples in case of DPT and TPT. In case of DPT and DPT, large number interacting residues which were alone were not covered. As shown above, the performance is better in case of DPT and TPT than SRT. The authors feel that one should used all three types of models for predicting GTP interacting residues rather than using individual method in order to achieve high accuracy.

## Conclusions

In this study, for the first time, a method has been developed to predict GTP interacting residues in a protein form its amino acid sequence with reasonably high accuracy. It has been observed that SVM models perform better than ANN based models in the prediction of GTP binding residues. The performance of models improved when PSSM profile has been used instead of single amino acid sequence of protein. First time, we have introduced and demonstrated that pattern could be used to predict more than one residue (e.g., dipeptide, tripeptides). The new models based on DPT and TPT perform better than standard models based on SRT. Though DPT and TPT improve performance but will decrease coverage so it is advisable to use all three models. Finally, web server has been developed which will serve the scientific community in understanding GTP-protein interaction.

## Methods

### Datasets

First we extracted 247 protein PDB IDs that interact with GTP from SuperSite encyclopedia [22]. We downloaded the sequence of all the chains of these PDB IDs from PDB. These sequences were filtered with 40% sequence identity using the program CD-HIT. Finally, we got 44 GTP binding chains, where no two sequences have more than 40% sequence identity. We used software Ligand Protein Contact (LPC) software [23] for assigning GTP-interacting and non-interacting residues in these 44 non-redundant protein chains.

### Pattern or window size

We generate overlapping patterns/segments/strings of size (or window size) 17. If the central residue of pattern is GTP interacting then we assigned the pattern as positive/interacting pattern otherwise pattern is assigned negative/non-interacting pattern [10,16]. To generate a pattern corresponding to the terminal residues in a protein sequence, we added (L-1)/2 dummy residue "X" at both termini of the protein (where L is the length of pattern). For window size 17 of SR patterns, we have added 8 "X" before N-terminal and 8 "X" after C-terminal, in order to create N patterns from the sequence of length N. We have generated a total 876 unique positive patterns from 44 protein chains. In this study, we have created two types of dataset - i) main dataset contains 876 positive patterns and equal number of randomly selected negative patterns; ii) realistic dataset contains 876 positive patterns and 16831 negative patterns. This is a standard technique for creating pattern around a single residue, almost of all the previous studies used this technique for developing method for predicting RNA-interacting residues [10], ATP-interacting residues [16], cleavage sites [24], signal peptides [25]. In this study, we called this a single residue based technique (SRT), as it is used to predict whether single central residue in pattern is interacting or non-interacting.

In this study, we have introduced two new techniques for predicting GTP interacting residues; i) dipeptide based technique (DPT) and ii) tripeptide based technique (TPT). In DPT, we have generated overlapping patterns/segments having interacting and non-interacting dipeptides (two consecutive residues) in center of peptides. In this case, positive patterns are those, which have two central residues GTP-interacting. Similarly, negative patterns are those, which have two central residues non-interacting (see Additional file 1 Figure S1). In TPT, we have generated overlapping patterns having interacting and non-interacting tripeptides (three consecutive residues) in center of peptides. In this case, the positive patterns are those, which have three central residues GTP-interacting. Similarly, the negative patterns are those, which have three central residues non-interacting (see Additional file 1 Figure S1).

### Five-fold cross-validation

In statistical prediction methods, there are three frequently used cross-validation techniques - single independent dataset test, sub-sampling (e. g., 5 or 10-fold cross-validation), test and jackknife test are widely used for examining the accuracy of a statistical prediction method [26,27]. In our study, we have used 5-fold cross-validation procedure to develop the prediction method, where five subsets have been constructed randomly from the data set as used in previous study [16]. Five-fold cross-validation is a popular cross-validation technique, which has no biasness in data selection. In this method, patterns are randomly divided into five sets. The methods have been trained on four sets, and the performance is measured on the remaining fifth set. This process is repeated five times in such a way that each set is used once for testing. The final performance is obtained by averaging the performances of all five sets.

## Artificial Neural Network

In this study, we have used SNNS (version 4.2 from) for implementing artificial neural network (ANN). This software is available free for academic use from Stuttgart University [28]. One of the beauties of this software is that it allows generating code in ANSI C for implementing neural models; this allows us to use in web-based implementation. The training is carried out using error back-propagation with a sum of square error function (SSE) [29]. The learning parameter was set to 0.01. The magnitude of the error sum in the test and training set was monitored after each cycle of training. Ultimately, the number of cycles is determined where the network during training converges.

### Support Vector Machine (SVM)

In this study, SVM_light has been used to implement SVM [30]. The SVM is a supervised machine-learning technique, based on the structural risk minimization principle from statistical learning theory. This package SVM_Light is freely available from http://www.cs.cornell.edu/People/ti/svm_light for academic use. Further detail about SVM can be obtained from Vapnik, 1995 [31]. The SVM_Light allows for choosing number of parameters and kernels (e.g. linear, polynomial, radial basis function, sigmoid) or any user-defined kernel.

### Amino acid Binary patterns

Assigning binary values to the amino acids in fixed length patterns generate amino acid binary patterns [10,16,32]. Previous studies on nucleotide interacting proteins prediction shows that 17 window patterns perform better than other window size [10,16]. A vector of dimension N × 21 represents the pattern of length N in binary form. Each residue was represented by a vector of dimension 21 (e.g. Ala by 1,0,0,0,0,0,0,0,0,0,0,0,0,0,0,0,0,0,0,0,0; Cys by 0,1,0,0,0,0,0,0,0,0,0,0,0,0,0,0,0,0,0,0,0), which contains 20 amino acids and one dummy amino acid "X".

### Position Specific Scoring Matrix (PSSM)

The PSSM profile for each protein was generated using PSI-BLAST by searching the protein against SWISS-PROT dataset [33]. In PSI-BLAST we use three iteration, after each iterative search in which sequences found in one round of searching are used to build a score model for the next round of searching. After three iterations with cut-off E-value of 0.001, PSI-BLAST generated a PSSM profile. The PSSM scores were normalized in order to get values between 0 and 1, and then position specific score of each amino acid was calculated. The matrix consisted of 21 × N elements (20 amino acids and one dummy amino acid "X"), where N is the length of the target sequence, and each element represents the frequency of occurrence of each of the 20 amino acids at one position in the alignment. It means that evolutionary information for each amino acid is encapsulated in a vector of 21 dimensions where the size of PSSM matrix of a protein with *N *residues is 21 × *N*. The resultant matrix with 357 elements for pattern of length 17-residue, was used as input feature for ANN or SVM.

### Evaluation Parameter

The performance of these prediction methods was evaluated by using standard parameter, which are routinely used. In current study the performance of all the methods and models was evaluated using 5-fold cross-validation using following equations.

1. Precision or probability of positive values (PPV) is the probability of correct prediction of interacting/positive residues.

2. Recall (R) is also called sensitivity or percent of coverage of GTP interacting residues in a protein. It is percentage of correctly predicted GTP-interacting residues in proteins.

3. F1 measure is the harmonic mean of precision (P) and recall (R).

(Precision, recall and the F1 measure are comprised between 0 and 1, the higher the value the better the performance)

4. Specificity is percentage of correctly predicted non-interacting residues in a protein.

5. Accuracy is the percentage of correctly predicted residues (interacting and non-interacting).

6. MCC - Matthews's correlation coefficient is the statistical parameter to access the quality of prediction and taking care of unbalancing in data. An MCC equal to 1 regarded as a perfect prediction, whereas 0 is for a completely random prediction.

7. AUC (Area under the ROC Curve) - Receiver Operating Curve (ROC) is a threshold independent parameter It is a plot between true positive proportion (TP/TP+FN) and false positive proportion (FP/FP+TN). We plot ROC and calculate AUC by using SPSS package.

Where, TP and TN are true positive (correctly predicted interacting) positive and true negative (correctly predicted non-interacting) residues respectively. FP and FN are false positive and false negatives respectively.

## Availability and requirements

Project name: GTPBinder: A server for prediction of GTP interacting protein residues

Project home page: http://www.imtech.res.in/raghava/gtpbinder

Operating system(s): Platform independent;

Programming language: PERL, CGI-PERL;

Other requirements: None;

License: None;

Any restrictions to use by non-academics: No restrictions.

## Competing interests

The authors declare that they have no competing interests.

## Authors' contributions

JSC created dataset and developed the SVM models. JSC, NKM created the backend web server and the front end user interface. GPSR conceived the project, coordinated it and refined the final manuscript drafted by JSC and NKM. All authors have read and approved final manuscript.

## Supplementary Material

Additional file 1 **Supplemental tables and figures**. Figure S1: Methodology of selection of different types of patterns (single GTP interacting residue, GTP interacting dipeptide and GTP interacting tripeptide). Table S1 to S6: The results of SVM on main dataset using different type of patterns. (40% sequence identity datasets). Table S7 to S12: The results of SVM on realistic dataset using different type of patterns. (40% sequence identity datasets). Click here for file
