# Supplementary material for: Prediction of GTP interacting residues, dipeptides and tripeptides in a protein from its evolutionary information
Source: BMC Bioinformatics. 2010 Jun 3;11:301. doi: 10.1186/1471-2105-11-301 (PMC3098072; doi:10.1186/1471-2105-11-301)
Supplement: Additional file 1 — Supplemental tables and figures. Figure S1: Methodology of selection of different types of patterns (single GTP interacting residue, GTP interacting dipeptide and GTP interacting tripeptide). Table S1 to S6: The results of SVM on main dataset using different type of patterns. (40% sequence identity datasets). Table S7 to S12: The results of SVM on realistic dataset using different type of patterns. (40% sequence identity datasets). [file 1471-2105-11-301-S1.DOC]

Additional file 1

Prediction of GTP-interacting residues, dipeptides and tripeptides in a protein from evolutionary information

Figure S1 Methodology of selection of different types of patterns used in this study. In single residue (SR) patterns single red or green color residue present in the center of 17 window length pattern and in dipeptide (DP) patterns center two consecutive red or green color residue present in the center of 16 window length while in tripeptide (TP) patterns three consecutive red or green color residue present in the center of 17 window length.


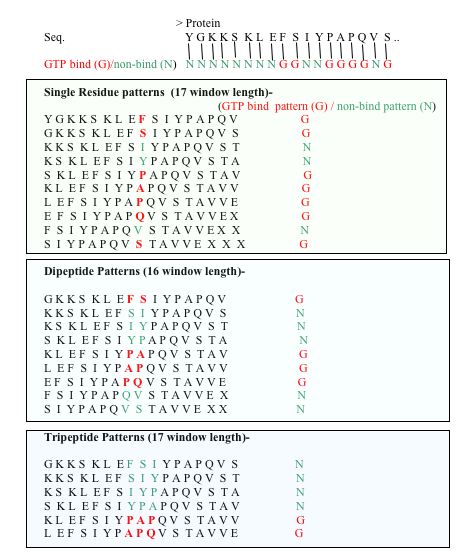


**Detail results on 40% sequence identity datasets (Table S1 to S12)**

**Performance on Main Dataset with 40% sequence identity**

Table S1. The Performance of SVM Model (Learning Parameter: g: 0.01 c: 1 j: 1) on Main Dataset Using Amino Acid Sequence Binary patterns where only one centre residue is GTP-interacting in fixed window length patterns. [g (in RBF kernel), c: parameter for trade-off between training error & margin, j: cost-factor]

(Bold values shows prediction results where MCC is maximum)

| **Threshold** | **Sensitivity (Recall)** | **Specificity** | **Accuracy** | **Precision** | **MCC** | **F1 Score** |
| --- | --- | --- | --- | --- | --- | --- |
| -1 | 97.6 | 8.56 | 53.08 | 0.52 | 0.14 | 0.68 |
| -0.9 | 96.69 | 11.42 | 54.05 | 0.52 | 0.16 | 0.68 |
| -0.8 | 95.32 | 14.84 | 55.08 | 0.53 | 0.17 | 0.68 |
| -0.7 | 93.84 | 20.78 | 57.31 | 0.54 | 0.21 | 0.69 |
| -0.6 | 89.5 | 29 | 59.25 | 0.56 | 0.23 | 0.69 |
| -0.5 | 85.62 | 35.84 | 60.73 | 0.57 | 0.25 | 0.69 |
| -0.4 | 82.19 | 42.58 | 62.39 | 0.59 | 0.27 | 0.69 |
| -0.3 | 77.74 | 50.91 | 64.33 | 0.61 | 0.30 | 0.69 |
| -0.2 | 72.03 | 57.19 | 64.61 | 0.63 | 0.30 | 0.67 |
| -0.1 | 67.35 | 65.07 | 66.21 | 0.66 | 0.32 | 0.67 |
| 0 | 62.67 | 72.6 | 67.64 | 0.70 | 0.35 | 0.66 |
| **0.1** | **57.19** | **78.77** | **67.98** | **0.73** | **0.37** | **0.64** |
| 0.2 | 49.66 | 83.56 | 66.61 | 0.75 | 0.35 | 0.6 |
| 0.3 | 42.92 | 87.1 | 65.01 | 0.77 | 0.33 | 0.55 |
| 0.4 | 35.84 | 90.07 | 62.96 | 0.78 | 0.31 | 0.49 |
| 0.5 | 28.77 | 93.38 | 61.07 | 0.81 | 0.29 | 0.42 |
| 0.6 | 24.66 | 95.09 | 59.87 | 0.83 | 0.28 | 0.38 |
| 0.7 | 20.78 | 97.03 | 58.9 | 0.88 | 0.28 | 0.34 |
| 0.8 | 15.98 | 97.6 | 56.79 | 0.87 | 0.24 | 0.27 |
| 0.9 | 12.44 | 98.4 | 55.42 | 0.89 | 0.21 | 0.22 |
| 1 | 9.93 | 98.74 | 54.34 | 0.89 | 0.19 | 0.18 |

Table S2. The Performance of SVM model (Learning Parameter: g: 0.01 c: 1 j: 1) on Main Dataset Using PSSM Patterns where only one centre residue is interacting.

| **Threshold** | **Sensitivity (Recall)** | **Specificity** | **Accuracy** | **Precision** | **MCC** | **F1 Score** |
| --- | --- | --- | --- | --- | --- | --- |
| -1 | 97.25 | 21.51 | 59.38 | 0.55 | 0.29 | 0.71 |
| -0.9 | 96.57 | 27.00 | 61.78 | 0.57 | 0.33 | 0.72 |
| -0.8 | 95.65 | 33.07 | 64.36 | 0.59 | 0.37 | 0.73 |
| -0.7 | 94.51 | 38.10 | 66.30 | 0.60 | 0.39 | 0.74 |
| -0.6 | 92.22 | 44.62 | 68.42 | 0.62 | 0.42 | 0.74 |
| -0.5 | 89.47 | 51.95 | 70.71 | 0.65 | 0.45 | 0.75 |
| -0.4 | 86.27 | 60.30 | 73.28 | 0.68 | 0.48 | 0.76 |
| -0.3 | 82.72 | 66.13 | 74.43 | 0.71 | 0.50 | 0.76 |
| -0.2 | 80.32 | 71.74 | 76.03 | 0.74 | 0.52 | 0.77 |
| -0.1 | 75.86 | 77.00 | 76.43 | 0.77 | 0.53 | 0.76 |
| **0** | **73.23** | **81.12** | **77.17** | **0.80** | **0.55** | **0.76** |
| 0.1 | 70.02 | 84.10 | 77.06 | 0.81 | 0.55 | 0.75 |
| 0.2 | 65.90 | 87.07 | 76.49 | 0.84 | 0.54 | 0.74 |
| 0.3 | 61.33 | 90.39 | 75.86 | 0.86 | 0.54 | 0.72 |
| 0.4 | 56.75 | 92.33 | 74.54 | 0.88 | 0.53 | 0.69 |
| 0.5 | 52.40 | 94.39 | 73.40 | 0.90 | 0.52 | 0.66 |
| 0.6 | 47.94 | 95.31 | 71.62 | 0.91 | 0.49 | 0.63 |
| 0.7 | 43.14 | 96.45 | 69.79 | 0.92 | 0.47 | 0.59 |
| 0.8 | 36.84 | 97.37 | 67.11 | 0.93 | 0.43 | 0.53 |
| 0.9 | 31.35 | 98.05 | 64.70 | 0.94 | 0.39 | 0.47 |
| 1 | 25.17 | 98.97 | 62.07 | 0.96 | 0.36 | 0.40 |

Table S3. The Performance of SVM model on Main dataset Using Amino acid Binary patterns, where centre two residue (Dipeptide) are interacting (g: 0.1 c: 3 j: 4)

| **Threshold** | **Sensitivity (Recall)** | **Specificity** | **Accuracy** | **Precision** | **MCC** | **F1 Score** |
| --- | --- | --- | --- | --- | --- | --- |
| -1 | 100.00 | 0.22 | 50.11 | 0.50 | 0.03 | 0.67 |
| -0.9 | 100.00 | 0.44 | 50.22 | 0.50 | 0.05 | 0.67 |
| -0.8 | 100.00 | 01.11 | 50.55 | 0.50 | 0.07 | 0.67 |
| -0.7 | 99.78 | 02.66 | 51.22 | 0.51 | 0.10 | 0.67 |
| -0.6 | 99.56 | 06.21 | 52.88 | 0.51 | 0.16 | 0.68 |
| -0.5 | 99.33 | 11.75 | 55.54 | 0.53 | 0.23 | 0.69 |
| -0.4 | 96.90 | 23.06 | 59.98 | 0.56 | 0.30 | 0.71 |
| -0.3 | 92.68 | 35.70 | 64.19 | 0.59 | 0.35 | 0.72 |
| -0.2 | 86.70 | 50.55 | 68.63 | 0.64 | 0.40 | 0.73 |
| -0.1 | 79.82 | 64.30 | 72.06 | 0.69 | 0.45 | 0.74 |
| 00 | 69.18 | 76.05 | 72.62 | 0.74 | 0.45 | 0.72 |
| **0.1** | **60.31** | **86.25** | **73.28** | **0.81** | **0.48** | **0.69** |
| 0.2 | 50.55 | 92.90 | 71.73 | 0.88 | 0.48 | 0.64 |
| 0.3 | 41.69 | 96.01 | 68.85 | 0.91 | 0.45 | 0.57 |
| 0.4 | 30.38 | 98.67 | 64.52 | 0.96 | 0.40 | 0.46 |
| 0.5 | 22.39 | 99.33 | 60.86 | 0.97 | 0.34 | 0.36 |
| 0.6 | 15.96 | 100.00 | 57.98 | 1.00 | 0.29 | 0.28 |
| 0.7 | 10.86 | 100.00 | 55.43 | 1.00 | 0.24 | 0.20 |
| 0.8 | 7.54 | 100.00 | 53.77 | 1.00 | 0.20 | 0.14 |
| 0.9 | 5.32 | 100.00 | 52.66 | 1.00 | 0.17 | 0.10 |
| 1 | 2.00 | 100.00 | 51.00 | 1.00 | 0.10 | 0.04 |

Table S4 The Performance of SVM model on Main dataset Using Amino acid PSSM patterns, where centre two residue (Dipeptide) are interacting (g: 0.01 c: 1 j: 1)

| **Threshold** | **Sensitivity (Recall)** | **Specificity** | **Accuracy** | **Precision** | **MCC** | **F1 Score** |
| --- | --- | --- | --- | --- | --- | --- |
| -1 | 98.89 | 21.51 | 60.2 | 0.56 | 0.32 | 0.71 |
| -0.9 | 98.23 | 27.27 | 62.75 | 0.57 | 0.36 | 0.73 |
| -0.8 | 96.01 | 32.82 | 64.41 | 0.59 | 0.37 | 0.73 |
| -0.7 | 95.34 | 39.91 | 67.63 | 0.61 | 0.42 | 0.75 |
| -0.6 | 93.13 | 47.23 | 70.18 | 0.64 | 0.45 | 0.76 |
| -0.5 | 89.58 | 53.88 | 71.73 | 0.66 | 0.47 | 0.76 |
| -0.4 | 87.58 | 63.19 | 75.39 | 0.7 | 0.52 | 0.78 |
| -0.3 | 85.81 | 70.73 | 78.27 | 0.75 | 0.57 | 0.8 |
| -0.2 | 83.37 | 74.72 | 79.05 | 0.77 | 0.58 | 0.8 |
| -0.1 | 79.6 | 78.94 | 79.27 | 0.79 | 0.59 | 0.79 |
| 0 | 77.16 | 84.92 | 81.04 | 0.84 | 0.62 | 0.8 |
| **0.1** | **73.61** | **89.14** | **81.37** | **0.87** | **0.64** | **0.8** |
| 0.2 | 67.63 | 92.02 | 79.82 | 0.89 | 0.62 | 0.77 |
| 0.3 | 64.08 | 95.34 | 79.71 | 0.93 | 0.63 | 0.76 |
| 0.4 | 61.64 | 96.45 | 79.05 | 0.95 | 0.62 | 0.75 |
| 0.5 | 58.09 | 97.12 | 77.61 | 0.95 | 0.60 | 0.72 |
| 0.6 | 53.22 | 97.78 | 75.5 | 0.96 | 0.57 | 0.68 |
| 0.7 | 48.56 | 98.67 | 73.61 | 0.97 | 0.55 | 0.65 |
| 0.8 | 41.24 | 99.33 | 70.29 | 0.98 | 0.5 | 0.58 |
| 0.9 | 35.92 | 99.33 | 67.63 | 0.98 | 0.46 | 0.53 |
| 1 | 29.49 | 99.33 | 64.41 | 0.98 | 0.4 | 0.45 |

Table S5. The Performance of SVM model on Main dataset Using Amino acid Binary patterns, where centre three residue (Tripeptide) are interacting (g: 0.1, c: 1,j: 1)

| **Threshold** | **Sensitivity (Recall)** | **Specificity** | **Accuracy** | **Precision** | **MCC** | **F1 Score** |
| --- | --- | --- | --- | --- | --- | --- |
| -1 | 100.00 | 0.00 | 50.00 | 0.50 | 0.00 | 0.67 |
| -0.9 | 100.00 | 0.00 | 50.00 | 0.50 | 0.00 | 0.67 |
| -0.8 | 100.00 | 0.00 | 50.00 | 0.50 | 0.00 | 0.67 |
| -0.7 | 100.00 | 0.39 | 50.20 | 0.50 | 0.04 | 0.67 |
| -0.6 | 100.00 | 01.56 | 50.78 | 0.50 | 0.09 | 0.67 |
| -0.5 | 100.00 | 07.03 | 53.52 | 0.52 | 0.19 | 0.68 |
| -0.4 | 99.22 | 14.84 | 57.03 | 0.54 | 0.26 | 0.70 |
| -0.3 | 94.14 | 30.86 | 62.50 | 0.58 | 0.32 | 0.72 |
| -0.2 | 86.33 | 52.73 | 69.53 | 0.65 | 0.41 | 0.74 |
| -0.1 | 75.78 | 77.34 | 76.56 | 0.77 | 0.53 | 0.76 |
| **0** | **62.5** | **89.06** | **75.78** | **0.85** | **0.53** | **0.72** |
| 0.1 | 53.91 | 96.09 | 75.00 | 0.93 | 0.55 | 0.68 |
| 0.2 | 43.36 | 99.22 | 71.29 | 0.98 | 0.51 | 0.60 |
| 0.3 | 35.16 | 100.00 | 67.58 | 1.00 | 0.46 | 0.52 |
| 0.4 | 27.34 | 100.00 | 63.67 | 1.00 | 0.40 | 0.43 |
| 0.5 | 18.36 | 100.00 | 59.18 | 1.00 | 0.32 | 0.31 |
| 0.6 | 10.16 | 100.00 | 55.08 | 1.00 | 0.23 | 0.18 |
| 0.7 | 05.86 | 100.00 | 52.93 | 1.00 | 0.17 | 0.11 |
| 0.8 | 03.12 | 100.00 | 51.56 | 1.00 | 0.13 | 0.06 |
| 0.9 | 01.17 | 100.00 | 50.59 | 1.00 | 0.08 | 0.02 |
| 1 | 0.39 | 100.00 | 50.20 | 1.00 | 0.04 | 0.01 |

Table S6: The Performance of SVM model on Main dataset Using Amino acid PSSM patterns, where centre three residue (Tripeptide) are interacting (g: 0.01,c: 2,j: 1)

| **Threshold** | **Sensitivity (Recall)** | **Specificity** | **Accuracy** | **Precision** | **MCC** | **F1 Score** |
| --- | --- | --- | --- | --- | --- | --- |
| -1 | 98.83 | 21.09 | 59.96 | 0.56 | 0.32 | 0.71 |
| -0.9 | 98.44 | 25.78 | 62.11 | 0.57 | 0.35 | 0.72 |
| -0.8 | 98.44 | 31.25 | 64.84 | 0.59 | 0.40 | 0.74 |
| -0.7 | 97.27 | 40.23 | 68.75 | 0.62 | 0.46 | 0.76 |
| -0.6 | 95.70 | 48.05 | 71.88 | 0.65 | 0.50 | 0.77 |
| -0.5 | 94.92 | 57.81 | 76.37 | 0.69 | 0.57 | 0.80 |
| -0.4 | 92.19 | 67.58 | 79.88 | 0.74 | 0.62 | 0.82 |
| -0.3 | 90.62 | 72.66 | 81.64 | 0.77 | 0.64 | 0.83 |
| -0.2 | 88.28 | 78.52 | 83.40 | 0.80 | 0.67 | 0.84 |
| -0.1 | 84.77 | 83.59 | 84.18 | 0.84 | 0.68 | 0.84 |
| 0 | 80.08 | 85.94 | 83.01 | 0.85 | 0.66 | 0.82 |
| 0.1 | 78.12 | 88.67 | 83.40 | 0.87 | 0.67 | 0.82 |
| 0.2 | 75.78 | 92.19 | 83.98 | 0.91 | 0.69 | 0.83 |
| **0.3** | **73.44** | **94.53** | **83.98** | **0.93** | **0.70** | **0.82** |
| 0.4 | 67.97 | 96.09 | 82.03 | 0.95 | 0.67 | 0.79 |
| 0.5 | 63.28 | 98.05 | 80.66 | 0.97 | 0.65 | 0.77 |
| 0.6 | 58.59 | 98.83 | 78.71 | 0.98 | 0.63 | 0.73 |
| 0.7 | 54.30 | 99.22 | 76.76 | 0.99 | 0.60 | 0.70 |
| 0.8 | 49.61 | 99.61 | 74.61 | 0.99 | 0.57 | 0.66 |
| 0.9 | 42.58 | 99.61 | 71.09 | 0.99 | 0.51 | 0.60 |
| 1 | 33.20 | 100 | 66.60 | 1.00 | 0.45 | 0.50 |

#### Performance on Realistic Dataset on 40% sequence identity

Table S7. The Performance of SVM model on Realistic dataset Using Amino acid Binary patterns, where centre one residue is GTP-interacting in fixed window length patterns. (g:0.01,c:8,j:4)

(Bold values shows prediction results where MCC is maximum)

| **Threshold** | **Sensitivity**  **(Recall)** | **Specificity** | **Accuracy** | **Precision** | **MCC** | **F1 Score** |
| --- | --- | --- | --- | --- | --- | --- |
| -1 | 0.71 | 67.28 | 67.49 | 0.10 | 0.18 | 0.18 |
| -0.9 | 0.64 | 76.76 | 76.13 | 0.13 | 0.20 | 0.21 |
| -0.8 | 0.57 | 84.37 | 83.01 | 0.16 | 0.23 | 0.25 |
| -0.7 | 0.49 | 89.84 | 87.84 | 0.20 | 0.26 | 0.29 |
| -0.6 | 0.43 | 93.64 | 91.13 | 0.26 | 0.29 | 0.32 |
| -0.5 | 0.37 | 96.21 | 93.27 | 0.34 | 0.32 | 0.35 |
| -0.4 | 0.31 | 97.71 | 94.42 | 0.41 | 0.33 | 0.36 |
| -0.3 | 0.27 | 98.7 | 95.15 | 0.52 | 0.35 | 0.36 |
| **-0.2** | **0.24** | **99.36** | **95.65** | **0.66** | **0.38** | **0.36** |
| -0.1 | 0.20 | 99.65 | 95.70 | 0.75 | 0.37 | 0.31 |
| 0 | 0.17 | 99.79 | 95.70 | 0.81 | 0.36 | 0.28 |
| 0.1 | 0.14 | 99.87 | 95.60 | 0.84 | 0.33 | 0.23 |
| 0.2 | 0.11 | 99.91 | 95.52 | 0.87 | 0.30 | 0.20 |
| 0.3 | 0.08 | 99.96 | 95.41 | 0.92 | 0.26 | 0.15 |
| 0.4 | 0.07 | 99.98 | 95.37 | 0.94 | 0.25 | 0.13 |
| 0.5 | 0.04 | 99.99 | 95.26 | 0.95 | 0.20 | 0.09 |
| 0.6 | 0.03 | 99.99 | 95.18 | 0.92 | 0.15 | 0.05 |
| 0.7 | 0.02 | 99.99 | 95.14 | 0.89 | 0.13 | 0.04 |
| 0.8 | 0.01 | 99.99 | 95.1 | 0.85 | 0.10 | 0.02 |
| 0.9 | 0.01 | 99.99 | 95.09 | 0.88 | 0.08 | 0.02 |
| 1 | 00 | 100 | 95.07 | 1.00 | 0.06 | 0.01 |

Table S8. The Performance of SVM model on Realistic dataset using PSSM patterns, where only centre residue is GTP-interacting. (g:0.01,c: 1,j: 4)

| **Threshold** | **Sensitivity (Recall)** | **Specificity** | **Accuracy** | **Precision** | **MCC** | **F1 Score** |
| --- | --- | --- | --- | --- | --- | --- |
| -1 | 78.03 | 72.13 | 72.44 | 0.13 | 0.24 | 0.23 |
| -0.9 | 71.74 | 81.18 | 80.7 | 0.17 | 0.28 | 0.28 |
| -0.8 | 67.51 | 87.49 | 86.46 | 0.23 | 0.34 | 0.34 |
| -0.7 | 63.16 | 92.03 | 90.54 | 0.3 | 0.39 | 0.41 |
| -0.6 | 58.47 | 94.91 | 93.04 | 0.38 | 0.44 | 0.46 |
| -0.5 | 53.89 | 96.77 | 94.57 | 0.47 | 0.48 | 0.50 |
| -0.4 | 50.11 | 97.81 | 95.36 | 0.55 | 0.50 | 0.53 |
| -0.3 | 47.83 | 98.51 | 95.9 | 0.63 | 0.53 | 0.55 |
| -0.2 | 46.00 | 99.01 | 96.28 | 0.72 | 0.56 | 0.56 |
| -0.1 | 44.16 | 99.37 | 96.53 | 0.79 | 0.58 | 0.57 |
| **0** | **41.65** | **99.55** | **96.57** | **0.83** | **0.57** | **0.56** |
| 0.1 | 39.59 | 99.68 | 96.6 | 0.87 | 0.57 | 0.54 |
| 0.2 | 37.76 | 99.76 | 96.58 | 0.90 | 0.57 | 0.53 |
| 0.3 | 35.24 | 99.81 | 96.49 | 0.91 | 0.55 | 0.51 |
| 0.4 | 33.07 | 99.86 | 96.43 | 0.93 | 0.54 | 0.49 |
| 0.5 | 30.09 | 99.89 | 96.31 | 0.94 | 0.52 | 0.46 |
| 0.6 | 28.03 | 99.95 | 96.26 | 0.97 | 0.51 | 0.43 |
| 0.7 | 25.06 | 99.98 | 96.13 | 0.98 | 0.49 | 0.40 |
| 0.8 | 20.94 | 99.98 | 95.91 | 0.98 | 0.44 | 0.34 |
| 0.9 | 15.56 | 99.99 | 95.65 | 0.99 | 0.38 | 0.27 |
| 1 | 10.07 | 99.99 | 95.37 | 0.98 | 0.31 | 0.18 |

Table S9. The Performance of SVM model on Realistic dataset using amino acid binary patterns, where centre two residues (Dipeptide) are GTP-interacting. (g: 0.01,c: 4 ,j: 3)

| **Threshold** | **Sensitivity (Recall)** | **Specificity** | **Accuracy** | **Precision** | **MCC** | **F1 Score** |
| --- | --- | --- | --- | --- | --- | --- |
| -1 | 77.83 | 70.88 | 71.52 | 0.21 | 0.30 | 0.33 |
| -0.9 | 74.5 | 74.57 | 74.56 | 0.23 | 0.31 | 0.35 |
| -0.8 | 71.4 | 78.41 | 77.77 | 0.25 | 0.33 | 0.37 |
| -0.7 | 69.18 | 81.77 | 80.61 | 0.28 | 0.35 | 0.40 |
| -0.6 | 65.41 | 84.42 | 82.68 | 0.30 | 0.36 | 0.41 |
| -0.5 | 62.31 | 87.13 | 84.86 | 0.33 | 0.38 | 0.43 |
| -0.4 | 59.87 | 89.68 | 86.95 | 0.37 | 0.40 | 0.46 |
| -0.3 | 54.99 | 91.96 | 88.57 | 0.41 | 0.41 | 0.47 |
| **-0.2** | **51.22** | **93.52** | **89.65** | **0.44** | **0.42** | **0.48** |
| -0.1 | 46.78 | 94.79 | 90.4 | 0.48 | 0.42 | 0.47 |
| 0 | 41.91 | 95.73 | 90.8 | 0.50 | 0.41 | 0.45 |
| 0.1 | 38.36 | 96.89 | 91.53 | 0.55 | 0.42 | 0.45 |
| 0.2 | 35.48 | 97.61 | 91.92 | 0.60 | 0.42 | 0.45 |
| 0.3 | 32.15 | 98.21 | 92.16 | 0.64 | 0.42 | 0.43 |
| 0.4 | 28.38 | 98.61 | 92.18 | 0.67 | 0.40 | 0.40 |
| 0.5 | 25.50 | 99.11 | 92.37 | 0.74 | 0.41 | 0.38 |
| 0.6 | 23.06 | 99.28 | 92.31 | 0.76 | 0.39 | 0.35 |
| 0.7 | 20.84 | 99.46 | 92.27 | 0.80 | 0.38 | 0.33 |
| 0.8 | 18.85 | 99.62 | 92.22 | 0.83 | 0.37 | 0.31 |
| 0.9 | 14.63 | 99.78 | 91.98 | 0.87 | 0.34 | 0.25 |
| 1 | 10.42 | 99.91 | 91.72 | 0.92 | 0.29 | 0.19 |

Table S10. The Performance of SVM model on Realistic dataset using amino acid PSSM patterns, where centre two residues (Dipeptide) are GTP-interacting (g: 0.01, c: 1 ,j: 4)

| **Threshold** | **Sensitivity (Recall)** | **Specificity** | **Accuracy** | **Precision** | **MCC** | **F1 Score** |
| --- | --- | --- | --- | --- | --- | --- |
| -1 | 90.24 | 67.76 | 69.8 | 0.22 | 0.34 | 0.35 |
| -0.9 | 87.14 | 74.14 | 75.32 | 0.25 | 0.38 | 0.39 |
| -0.8 | 84.04 | 80.19 | 80.54 | 0.30 | 0.42 | 0.44 |
| -0.7 | 81.37 | 85.22 | 84.87 | 0.35 | 0.47 | 0.49 |
| -0.6 | 77.38 | 89.14 | 88.07 | 0.42 | 0.51 | 0.54 |
| -0.5 | 75.17 | 92.00 | 90.47 | 0.48 | 0.55 | 0.59 |
| -0.4 | 71.40 | 94.28 | 92.20 | 0.56 | 0.59 | 0.62 |
| -0.3 | 68.96 | 95.79 | 93.35 | 0.62 | 0.62 | 0.65 |
| -0.2 | 66.52 | 97.03 | 94.26 | 0.69 | 0.65 | 0.68 |
| -0.1 | 64.30 | 98.01 | 94.94 | 0.76 | 0.67 | 0.70 |
| **0** | **62.31** | **98.63** | **95.33** | **0.82** | **0.69** | **0.71** |
| 0.1 | 59.87 | 99.00 | 95.45 | 0.86 | 0.69 | 0.70 |
| 0.2 | 56.76 | 99.34 | 95.47 | 0.90 | 0.69 | 0.69 |
| 0.3 | 54.10 | 99.58 | 95.45 | 0.93 | 0.69 | 0.68 |
| 0.4 | 51.22 | 99.69 | 95.29 | 0.94 | 0.68 | 0.66 |
| 0.5 | 48.34 | 99.78 | 95.10 | 0.96 | 0.66 | 0.64 |
| 0.6 | 43.90 | 99.87 | 94.78 | 0.97 | 0.63 | 0.60 |
| 0.7 | 38.36 | 99.96 | 94.36 | 0.99 | 0.60 | 0.55 |
| 0.8 | 33.70 | 99.98 | 93.96 | 0.99 | 0.56 | 0.50 |
| 0.9 | 27.27 | 99.98 | 93.37 | 0.99 | 0.50 | 0.43 |
| 1 | 24.17 | 99.98 | 93.09 | 0.99 | 0.47 | 0.39 |

Table S11. The Performance of SVM model on Realistic dataset using amino acid binary patterns, where centre Three residues (Tripeptide) are GTP-interacting (g: 0.01, c: 10, j: 2)

| **Threshold** | **Sensitivity (Recall)** | **Specificity** | **Accuracy** | **Precision** | **MCC** | **F1 Score** |
| --- | --- | --- | --- | --- | --- | --- |
| -1 | 79.30 | 75.00 | 75.39 | 0.24 | 0.34 | 0.37 |
| -0.9 | 76.17 | 78.61 | 78.39 | 0.26 | 0.36 | 0.39 |
| -0.8 | 72.27 | 82.34 | 81.43 | 0.29 | 0.37 | 0.41 |
| -0.7 | 69.53 | 84.94 | 83.55 | 0.31 | 0.39 | 0.43 |
| -0.6 | 65.23 | 87.38 | 85.38 | 0.34 | 0.40 | 0.45 |
| -0.5 | 60.55 | 89.67 | 87.04 | 0.37 | 0.40 | 0.46 |
| -0.4 | 56.64 | 91.96 | 88.77 | 0.41 | 0.42 | 0.48 |
| -0.3 | 54.30 | 93.44 | 89.90 | 0.45 | 0.44 | 0.49 |
| **-0.2** | **50.39** | **94.60** | **90.61** | **0.48** | **0.44** | **0.49** |
| -0.1 | 46.09 | 95.61 | 91.14 | 0.51 | 0.44 | 0.48 |
| 0 | 42.97 | 96.39 | 91.56 | 0.54 | 0.44 | 0.48 |
| 0.1 | 39.45 | 97.20 | 91.98 | 0.58 | 0.44 | 0.47 |
| 0.2 | 37.89 | 97.83 | 92.41 | 0.63 | 0.45 | 0.47 |
| 0.3 | 33.59 | 98.25 | 92.41 | 0.66 | 0.43 | 0.44 |
| 0.4 | 31.25 | 98.76 | 92.66 | 0.71 | 0.44 | 0.43 |
| 0.5 | 27.34 | 99.11 | 92.62 | 0.75 | 0.43 | 0.40 |
| 0.6 | 21.48 | 99.38 | 92.34 | 0.77 | 0.38 | 0.34 |
| 0.7 | 18.36 | 99.50 | 92.16 | 0.78 | 0.36 | 0.30 |
| 0.8 | 14.45 | 99.61 | 91.91 | 0.79 | 0.32 | 0.24 |
| 0.9 | 10.55 | 99.69 | 91.63 | 0.77 | 0.27 | 0.19 |
| 1 | 09.77 | 99.77 | 91.63 | 0.81 | 0.26 | 0.17 |

Table S12. The Performance of SVM model on Realistic dataset using amino acid PSSM patterns, where centre three residues (Tripeptide) are GTP-interacting. (g: 0.01, c: 2, j: 2)

| **Threshold** | **Sensitivity (Recall)** | **Specificity** | **Accuracy** | **Precision** | **MCC** | **F1 Score** |
| --- | --- | --- | --- | --- | --- | --- |
| -1 | 88.28 | 71.81 | 73.24 | 0.23 | 0.36 | 0.36 |
| -0.9 | 86.33 | 78.94 | 79.58 | 0.28 | 0.42 | 0.42 |
| -0.8 | 84.77 | 84.99 | 84.97 | 0.35 | 0.48 | 0.49 |
| -0.7 | 80.47 | 89.00 | 88.26 | 0.41 | 0.52 | 0.54 |
| -0.6 | 77.34 | 92.38 | 91.08 | 0.49 | 0.57 | 0.60 |
| -0.5 | 76.17 | 95.13 | 93.49 | 0.60 | 0.64 | 0.67 |
| -0.4 | 74.22 | 96.73 | 94.78 | 0.68 | 0.68 | 0.71 |
| -0.3 | 71.88 | 97.81 | 95.56 | 0.76 | 0.71 | 0.74 |
| -0.2 | 68.36 | 98.48 | 95.86 | 0.81 | 0.72 | 0.74 |
| **-0.1** | **66.80** | **99.11** | **96.30** | **0.88** | **0.75** | **0.76** |
| 0 | 63.28 | 99.52 | 96.37 | 0.93 | 0.75 | 0.75 |
| 0.1 | 60.94 | 99.70 | 96.34 | 0.95 | 0.75 | 0.74 |
| 0.2 | 58.59 | 99.74 | 96.17 | 0.96 | 0.73 | 0.73 |
| 0.3 | 55.47 | 99.81 | 95.96 | 0.97 | 0.72 | 0.70 |
| 0.4 | 51.95 | 99.93 | 95.76 | 0.99 | 0.70 | 0.68 |
| 0.5 | 51.17 | 99.93 | 95.69 | 0.98 | 0.69 | 0.67 |
| 0.6 | 47.66 | 99.96 | 95.42 | 0.99 | 0.67 | 0.64 |
| 0.7 | 43.36 | 99.96 | 95.05 | 0.99 | 0.64 | 0.60 |
| 0.8 | 39.45 | 100.00 | 94.74 | 1.00 | 0.61 | 0.57 |
| 0.9 | 32.81 | 100.00 | 94.17 | 1.00 | 0.56 | 0.49 |
| 1 | 22.66 | 100.00 | 93.28 | 1.00 | 0.46 | 0.37 |
